# International Infectious Disease Society for Obstetrics and Gynecology–USA

**DOI:** 10.1155/S1064744996000233

**Published:** 1996

**Authors:** William J. Ledger, Mark G. Martens

**Affiliations:** Department of Obstetcs and Gynecology New York Hospital/Cornell Medical Center New York New York USA


					Infectious Diseases in Obstetrics and Gynecology 4:104 (1996)
(C) 1996 Wiley-Liss, Inc.
International Infectious Disease Society for
Obstetrics and Gynecology-USA
In the summer and fall of 199.5, the International Infectious Disease Society for
Obstetrics and Gynecology-USA was founded, with a commitment to the discipline
of infectious disease and the health care of women worldwide. Scientists from other
corners ofthe world have different clusters ofdiseases, different laboratory approaches,
and new methods of problem solving that often shed light on unresolved questions
regarding society. By establishing this organization, the founding members have
succeeded in their goal to share knowledge through freer access to infectious disease
specialists around the world.
Published in this issue are the abstracts of the first meeting of the International
Infectious Disease Society for Obstetrics and Gynecology-USA held in Boulder, CO,
on April 27-29, 1996. While the readers can judge for themselves the quality of the
abstracts, what cannot be conveyed in writing are the zest of the discussions and the
camaraderie ofthose attending the meeting. The American and international members
as well as guests were enthusiastic about our new organization and plans for the next
meeting in Nevada in the spring of 1997. We share their anticipation of an exciting
and stimulating event.
William J. Ledger, President
Mark G. Martens, 1996 Annual Meeting Program Chairman
Department ofObstetcs and Gynecology
New York Hospital/Cornell Medical Center
New York, New York
Editorial
Received July 3, 1996
Accepted July 8, 1996

				

